# The Dynamic Network of RNP RNase P Subunits

**DOI:** 10.3390/ijms221910307

**Published:** 2021-09-24

**Authors:** Athanasios-Nasir Shaukat, Eleni G. Kaliatsi, Ilias Skeparnias, Constantinos Stathopoulos

**Affiliations:** Department of Biochemistry, School of Medicine, University of Patras, 26504 Patras, Greece; a.sokat@upnet.gr (A.-N.S.); e_kaliatsi@upatras.gr (E.G.K.); iskeparnias@upatras.gr (I.S.)

**Keywords:** RNase P, pre-tRNA, RNPs, Rpps, ribozyme

## Abstract

Ribonuclease P (RNase P) is an important ribonucleoprotein (RNP), responsible for the maturation of the 5′ end of precursor tRNAs (pre-tRNAs). In all organisms, the cleavage activity of a single phosphodiester bond adjacent to the first nucleotide of the acceptor stem is indispensable for cell viability and lies within an essential catalytic RNA subunit. Although RNase P is a ribozyme, its kinetic efficiency in vivo, as well as its structural variability and complexity throughout evolution, requires the presence of one protein subunit in bacteria to several protein partners in archaea and eukaryotes. Moreover, the existence of protein-only RNase P (PRORP) enzymes in several organisms and organelles suggests a more complex evolutionary timeline than previously thought. Recent detailed structures of bacterial, archaeal, human and mitochondrial RNase P complexes suggest that, although apparently dissimilar enzymes, they all recognize pre-tRNAs through conserved interactions. Interestingly, individual protein subunits of the human nuclear and mitochondrial holoenzymes have additional functions and contribute to a dynamic network of elaborate interactions and cellular processes. Herein, we summarize the role of each RNase P subunit with a focus on the human nuclear RNP and its putative role in flawless gene expression in light of recent structural studies.

## 1. The Discovery of Different RNase P Facets

Ribonuclease P (RNase P) is an essential endoribonuclease that was first characterized by Sidney Altman almost 40 years ago in *E. coli*, as a complex of one protein subunit (RnpA or C5 protein; 119 aa; 13,789 Da) and an RNA subunit (M1 RNA; 377 nt). Surprisingly, the RNA subunit was found to perform standalone catalysis in vitro, paving the way for the discovery of ribozymes and the contemporary RNA biology [1]. RNase P is responsible for the first and most important step of tRNA biogenesis in all organisms. It matures the 5′ end of pre-tRNAs through a single and accurate endonucleolytic cleavage adjacent to the first base-pair of the acceptor stem. In prokaryotes and organelles, RNase P liberates tRNAs from longer primary transcripts that may also include rRNAs, and in eukaryotes, it processes precursor transcripts of individual tRNA genes bearing 5′ leader and 3′ trailer sequences [2].

During a long period of studies on RNase P, the emerged remarkable structural and functional variability of the holoenzyme among different organisms surprised and puzzled the field, raising questions on the evolution of catalytic RNAs and the transition events from an RNA world to the contemporary RNP and protein world. More importantly, although several breakthrough studies have deciphered the catalytic mechanism and the role of important co-factors, such as the divalent metal ions on the active site, a complete picture of the holoenzyme structures remained elusive until very recently [3,4,5]. Although the bacterial RNase P holoenzyme consists of a single RNA subunit and one rather small protein, studies on RNase P from several archaea showed a more complex ribonucleoprotein, of one RNase P RNA (RPR) and five protein subunits (Pop5, Rpp21, Rpp29, Rpp30 and L7Ae/Rpp38) [6]. At the same time, the RNase P from yeast and humans exhibit a more elaborate structural organization. In all eukaryotes, the RNase P holoenzyme is more proteinaceous and includes one RNA and several protein subunits (Table 1).

In humans, the RNA subunit (*RPPH1*; Ribonuclease P RNA Component H1) is wrapped by eleven polypeptides (Rpp14, Pop5, Rpp20, Rpp21, Rpp25, Rpp29, two copies of Rpp30, Rpp38, Rpp40, and Pop1) and is the largest RNase P RNP that has been described to date (Table 1). Of note, two *RPPH1* paralogue putative pseudogenes are annotated in the human genome (*RPPH1-2P*; 493 nt and *RPPH1-3P*; 522 nt, RefSeq Nos. NG_043330 and NG_044828, respectively). Interestingly, three similar paralogues have been reported differentially expressed in various murine tissues (Rprl1-3; 238, 238, 235 nt, respectively; RefSeq Nos. NR_004434.3, NR_004439.2, and NR_024198.3, respectively), raising more questions on the uniform composition of the holoenzyme in mammals [7].

Since the early days of RNase P studies, distinct 5′ tRNA maturation activities have been also detected in chloroplasts and mitochondria. However, these activities seem to be independent of a catalytic RNA subunit, suggesting the existence of holoenzymes that either lost their RNA subunit, or were protein-only enzymes [8,9]. Surprisingly, the human mitochondrial RNase P (mt-RNase P) was found to deviate from the typical ribonucleoprotein pattern and is a protein-only enzyme (termed PRORP) of three subunits, which are encoded in the nuclear genome (MRPP1, MRPP2, and MRPP3) [9]. MRPP3 is the catalytic subunit of mt-RNase P and requires the binding of MRPP1/2 complex to process the 5′ of mitochondrial pre-tRNAs [10]. Based on the previous, three MRPP3 orthologues (PRORP1-3) were also found in plants [11]. PRORP1 is localized in the mitochondria and chloroplasts of *A. thaliana,* where it exhibits an essential RNase P activity and acts as a single subunit. On the other hand, PRORP2 and PRORP3 are targeted to the nucleus, where they are responsible in pre-tRNA processing [11]. Moreover, homologues of Rpps that were identified in *A. thaliana* seem to be dedicated to their role in the context of RNase MRP (mitochondrial RNA Processing) [12,13]. Although PRORPs were thought to exist only in eukaryotes, later studies identified a protein-only RNase P enzyme initially in the bacterium *A. aeolicus* and later in other prokaryotes, and these enzymes were named HARPs (homologs of *Aquifex aeolicus* protein-only RNase P) [14,15]. Interestingly, some bacteria, including *A. aeolicus*, do not encode an RNase P RNP, suggesting divergent roles for each enzyme [15].

Along the way, studies on the complexity of RNase P composition were enriched by analyses regarding the substrate specificity and the role of RNase P on processing additional substrates, apart from tRNAs. For example, RNase P can cleave multiple substrates such as the 4.5S RNA (in *E. coli*) and long non-coding RNAs (lncRNAs), such as *MALAT1* and *MEN-β/NEAT1*, which contain tRNA lookalike structures [16,17,18]. In addition, several studies have shown that the protein subunits of the holoenzyme play additional roles in important cellular processes, spanning from chromatin organization to transcription and translation regulation, either as individual components or as part of larger complexes [19,20]. For many years, the multiple facets of RNase P RNPs called for in-depth structural analysis and raised questions on the evolution of different structures and functions that primarily and predominantly serve the biogenesis of mature tRNAs as substrates of translation. Today, the available structural information from bacteria, archaea and human RNase P RNP complexes, in comparison with structural data from the mitochondrial PRORP, suggest a conserved structural rigidity of RNase P, which allows this multiple-turnover enzyme to process thousands of substrates by acting as a molecular caliper for the tRNA-elbow/3′ end axis, which is considered the “ancient” part of the tRNA [21]. In addition, it has been suggested that the catalytic RNA subunit of RNase P has evolved from a true ribozyme to the RNA core of the RNP complex, which allowed the acquired protein subunits of eukaryotic RNase P RNPs to sustain or develop additional roles in cellular metabolism.

## 2. Universal Recognition of pre-tRNAs by RNase P Holoenzymes from a Structural Point of View

The elucidation of the holoenzymes’ architecture was long considered a holy grail, due to limitations in the crystallization of large complexes. The first crystal structure of RNase P from the thermophile bacterium *Thermotoga maritima* was made available 10 years ago and shed light on the binding mode of pre-tRNA^Phe^ and the catalytic mechanism [3]. The *T. maritima* RNase P RNA is a Type-A subunit (P RNA) very similar to its *E. coli* counterpart (M1 RNA). The RNase P RNA-tRNA interactions in the crystal structure confirmed previous biochemical studies, where M1 RNA and tRNAs interacted (i) via base stacking of the CR-II (conserved region II), CR-III and P11 helix with the D-loop and T-loop (G/U19 and C56, respectively), (ii) with an A-minor interaction between P4 (which is in the C domain) and the acceptor stem, and (iii) via the formation of canonical base pairs at the 3′ CCA with the L15 [3]. Although pre-tRNA^Phe^ was used as the substrate for structure determination, only the mature tRNA was detected in the crystals, due to the cleavage by RNase P, and a short oligonucleotide was used for the necessary detection of possible interactions with the 5′ leader of pre-tRNA. The structure showed that the 5′ leader unwinds through interactions of nucleotides toward its 5′ end with the protein component, while nucleotides 1 to 3 interact with the P RNA. It should be noted that although the pre-tRNA^Phe^ used in the study encoded the 3′ CCA end, some bacteria encode CCA-less tRNAs, and therefore, the reported interaction with the CCA is considered important but not essential as was initially observed [22]. In that respect, the structural analysis of catalytically inactive RNase P mutants with a CCA-less pre-tRNA could provide useful information.

The archaeal and eukaryotic RNase P holoenzymes retained their RNA subunits as catalysts, albeit with slower turnover rates and aberrant cleavage sites [23,24,25,26,27]. The enrichment in protein content, although attributed to evolutionary events, was puzzling until the recent, detailed Cryo-EM structure of human nuclear RNase P holoenzyme was presented [4]. Although several previous studies on the reconstitution of the archaeal and eukaryotic enzymes provided an outline of the RNA–protein and protein–protein interactions, the structure of the human nuclear RNase P, both in its apo form and in complex form with tRNA^Val^, provided the detailed architecture of the RNP and highlighted the importance of protein subunits in the assembly and the function of the holoenzyme. Interestingly, the structure revealed a hand-shaped protein clamp tightly wrapped around the *RPPH1* RNA subunit, which in turn interacts with all the protein components. Protein subcomplexes are also observed in the context of the holoenzyme (Rpp20-Rpp25, Pop5-Rpp14-(Rpp30)_2_-Rpp40, Rpp21-Rpp29-Rpp38, and a single Pop1 polypeptide) that act as a scaffold to stabilize the RNA subunit in an optimal conformation for tRNA binding [4]. Recognition of the tRNA’s “elbow” by *RPPH1* is achieved through interactions of the T- (C56) and D- loops (U19) with the CR-II and CR-III modules of the S domain of *RPPH1*, similar to what was described for bacterial RPR [3]. However, the anchoring of the acceptor stem and its coordination to the active site (stem P4) is mediated by Pop1. As in the case of the bacterial RNase P structure, the 5′ leader of pre-tRNA (tRNA^Val^) is not present in the crystal structure; however, given the placement of Pop5 in the holoenzyme, it is possible that this subunit interacts with the 5′ leader in the same manner as the bacterial Rpp. The human pre-tRNA^Val^ contained additionally a 3′ trailer, like most of the eukaryotic pre-tRNA transcripts, which, upon transcription, are protected by the La protein [28]. The 3′ trailer does not form any detectable interactions with the complex, suggesting that the absence of 3′ CCA ends in eukaryotic pre-tRNAs does not affect recognition and interaction with the holoenzyme.

The subsequent Cryo-EM structure of RNase P from the archaeon *Methanocaldococcus jannaschii* completed the picture of the RNase P RNP variants. In archaea, three distinct types of RPRs are found, namely, the A-type (very similar to the bacterial RPR), the M-type and the P-type [29,30,31]. The A-type and M-type exhibit ribozyme activity in vitro, with the A-type being a very weak catalyst [32]. The structure of *M. jannaschii* RNase P (M-type) indicates that the archaeal holoenzyme adopts a rigid dimeric conformation with two-form symmetry, containing 10 protein subunits and 2 catalytic RPRs [5]. The protein subunits consist of one (Pop5-Rpp30)_2_ heterotetramer and two Rpp29-Rpp21-L7Ae heterotrimers forming a long decamer. The interactions formed between the tRNA and the RPR in archaea are very similar to that of bacteria, with the T- and D-loops of the pre-tRNA directly interacting with CR-II and CR-III of the S domain, while the acceptor stem interacts with a three-nucleotide linker between the P5 and P15 of the C domain. Thus, the archaeal RNase P contains two RNA-based anchors for substrate recognition. The equivalent of the bacterial RPR L15 that interacts with the 3′ CCA of the tRNA is missing in *M. jannaschii*. This observation coincides with what was reported for the human RNase P structure, as discussed above.

**Figure 1:**
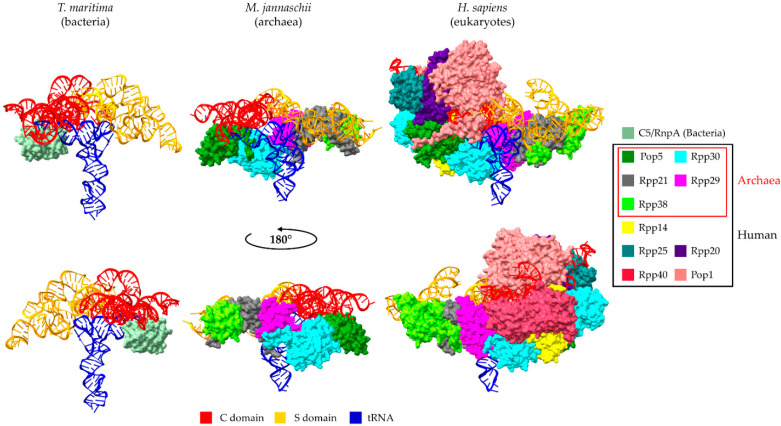
Comparison of the RNase P structures, in complex with pre-tRNA from *T. maritima* (PDB ID: 3Q1R), *M. jannaschii* (PDB ID: 6K0B, edited to show the monomeric RNase P) and human (PDB ID: 6AHU). The structures were visualized using ChimeraX v1.2 [35].

The knowledge of detailed RNase P RNP structures from bacteria, archaea and eukaryotes allows a direct comparison and better understanding of tRNA recognition (Figure 1). From bacteria to archaea, the ribozyme maintained the catalytic core of the RNA subunit (C domain) but lost a key element in the specificity domain (S domain), which stabilizes the distance between the two tRNA-binding anchors in bacterial RNase Ps [5]. In addition, the P12 stem evolved into a double-L-shaped structure with a K-turn that is recognized by the newly acquired protein Rpp38, suggesting that the archaeal RNase P still maintains two RNA-based anchors for substrate binding, but the stabilization mechanism of the anchors evolved into one that requires a complicated protein complex. The human *RPPH1* is shaped into an extended and single-layered conformation, compared to the compact and double-layered form that bacterial RNAs adopt [4]. In addition, although it has lost important elements, which are present in bacterial and most of the archaea (such as P6, P15 and P16), it retains conserved core domains (such as CR-II and CR-III), which ensures RNA-based catalysis. However, the subunit responsible for the anchoring of the tRNA’s acceptor stem is Pop1 protein and not *RPPH1* RNA. RNase P anchors the tRNA “elbow” and the acceptor stem and acts as a molecular ruler to correctly process pre-tRNAs and to recognize mistranscribed molecules. It should be noted that this type of interaction is also observed in additional, high-ordered RNA–RNA interactions, like in the case of T-box riboswitches, and represents a conserved mode of tRNA recognition [21,33]. Since this review is focused on Rpps and not the RNA subunits, we refer to a recent review by Phan and colleagues for a structural comparison of the RPRs [34].

Interestingly, structural and biochemical studies on plant PRORPs showed that tRNA recognition is similar to that by RNase P RNP and acts as single protein enzymes [36,37,38]. The structure of PRORP1 and -2 from *A. thaliana* revealed that this enzyme consists of two domains, the PPR and the NYN domains, which are responsible for tRNA binding and catalysis, respectively [36,37]. Further biochemical experiments and structural analysis of the PPR domain in complex with tRNA revealed that PRORP1 interacts with the D- and T-loops of tRNAs, similar to that by RPRs, the 23S rRNA and T-box riboswitches [39,40]. Moreover, small angle X-ray scattering of *A. thaliana* PRORP1 and PRORP2 in complex with tRNA showed that the tRNA is clamped by these two PRORPs similarly to the bacterial RPRs and that the 5′ end is located inside the NYN-domain, in close proximity to the zinc-binding site [38,39,41]. Moreover, the protein-only HARPs found in prokaryotes are smaller enzymes than plant PRORPs and lack a PPR-equivalent domain [15]. As shown by the recent structures from *A. aeolicus* and *H. halophila*, HARPs form homododecamers with two adjacent subunits required for processing a single pre-tRNA [42,43]. Recognition of pre-tRNAs by HARPs is achieved through a manner similar to plant PRORPs and RNase P RNP [42].

Recently, the complete structure of the human mt-RNase P in complex with a mitochondrial tRNA was determined; it forms a heterohexamer (MRPP3-MRPP1-(MRPP2)_4_), revealing the absence of interaction with the tRNA’s D-loop [44]. MRPP3, which is homologous to PRORP1 and PRORP2, requires the presence of MRPP1 and MRPP2 for catalysis. Comparison of the known PRORP1 and MRPP3 structures revealed that, even though both proteins have almost identical conformation, the sub-domains are disordered and MRPP3 cannot form a divalent–cation binding site and thus, cannot catalyze RNA hydrolysis without the contribution of MRPP1 and MRPP2 in the overall conformation [45].

## 3. The Protein Subunits of Eukaryotic RNase P RNPs and Their Dynamic Networks

RNase P represents an excellent example of molecular evolution, which allows primordial RNA catalysts to shape the essential contemporary RNPs, such as ribosome, spliceosome and telomerase. Although, the RNA subunit in all species is responsible for catalysis, the existence of multiple RNase P protein subunits raises important questions regarding the additional roles of these proteins in cell physiology. Several studies have shown that the protein subunits can dissociate from the holoenzyme to participate in cellular processes, such as transcription, DNA damage response (DDR) and chromatin assembly, independently or in coordination with other RNase P subunits (Table 2) [20]. These alternative roles are summarized below.

### 3.1. Rpp14

**Figure 2:**
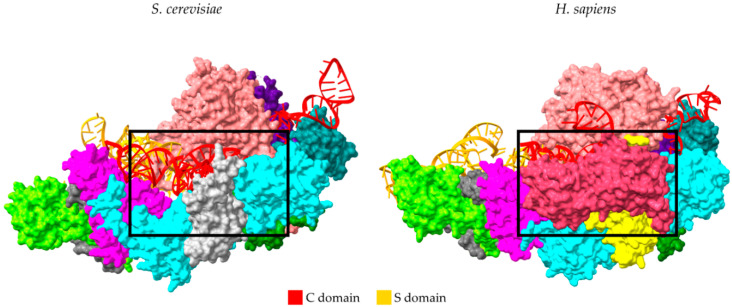
Comparison of Cryo-EM structures of yeast (**left**, PDB ID: 6AGB) and human RNase P holoenzymes (**right**, PDB ID: 6AHR). The human holoenzyme has lost Pop8 found in yeast (light grey) and acquired Rpp14 (yellow) and hRpp40 (red). The black square indicates the corresponding site of human Rpp40 on the yeast holoenzyme. The structures were visualized using ChimeraX v1.2 [35].

The human *RPP14* gene is mapped to chromosome 3p14.3, and the protein is produced by a bi-cistronic transcript, which also produces the HTD2 protein (hydroxyacyl-thioester dehydratase type 2) from a downstream open reading frame. hRpp14 is localized in nucleoplasm and also in the dense fibrillar component of nucleoli [57]. The lack of a nucleolar localization sequence in Rpp14 suggests the dependency on a “piggyback” mechanism for translocation into the nucleolus [58]. A homologue of Rpp14 is absent in archaeal and yeast holoenzymes, although archaeal Pop5 is considered the ancestor of both eukaryotic Pop5 and Rpp14 [4,5,59]. Consequently, the archaeal (Pop5-Rpp30)_2_ heterotetramer, which was observed in the Cryo-EM structure, is considered equivalent to the Pop5-Rpp14-(Rpp30)_2_ observed heterotetramer in humans, and the binding site of Rpp14 is similar to that of Pop8 in yeast (Figure 2).

Two-hybrid system screening showed that hRpp14 interacts with OIP2/EXOSC8 (opacity-associated protein 2/Exosome component 8) and has a 3′→5′ exoribonucleases activity in vitro, which suggests a possible role in RNA turnover [46,60]. Although OIP2 is not well characterized, it is considered a component of the RNA exosome complex. However, it was found that OIP2, as well as Rpp14, contains an RNase PH domain, which has an affinity for U- and AU-rich sequences [61]. Moreover, Rpp14 binds to LIMD1 (LIM domain-containing protein 1), which belongs to a DNA-binding family. LIMD1 is involved in numerous cellular processes, including cytoskeletal organization, repression of gene transcription, cell differentiation, proliferation and migration, and the miRNA pathway. However, further studies are required to assess the significance of this interaction [60,62].

### 3.2. Pop5

Pop5 protein is widely distributed among organisms, and its homologues are very similar in size. However, fungal Pop5 has an insertion of ∼25 amino acids in length, close to the N-terminal end [63]. In humans, the *POP5* gene is mapped to chromosome 12q24.31, and hPop5 is localized in the nucleus and also in the nucleolus [64]. It was reported that hPop5 does not contain a nuclear localization signal and is transported to the nucleus and nucleolus by a “piggyback” mechanism. hPop5 possibly binds to another RNase P protein in the cytoplasm, which carries the Pop5 into the nucleus and subsequently to the nucleolus. The transportation of the Pop5 in the nucleolus may be dependent on its association with the partially assembled RNase MRP/RNase P ribonucleoprotein complexes [64].

Although the archaeal and eukaryal Pop5s are not considered homologs of the bacterial protein component, they bind to the C domain of the RNA component in a similar region. Moreover, both Pop5 and the bacterial protein interact and stabilize the 5′ leader of pre-tRNAs. In the context of the human holoenzyme, hPop5 is part of the “palm” module, which is comprised of the heteropentamer Pop5-(Rpp30)_2_-Rpp14-Rpp40 [3,4].

### 3.3. Rpp20

In humans, the *RPP20* gene is mapped to chromosome 7q22.1 and encodes a protein, which belongs to Alba (Acetylation lowers binding affinity)–like proteins and is characterized as an ATPase, after purification of HeLa nuclear RNase P [47]. Although RNase P does not require ATPase activity to cleave its substrates, it is likely that Rpp20 uses this activity for other functions, such as chromatin remodeling. Later studies showed that Rpp20 (but also Rpp25) binds to the chromatin of active rDNA genes (discussed below) [49]. hRpp20 is localized primarily in nucleoli after heterodimerization with hRpp25 [65]. The yeast homologue of Rpp20, known as Rpp2 or Pop7, represents an essential protein in *S. cerevisiae*. In vivo experiments with a conditional lethal yeast strain of Rpp2 showed an accumulation of pre-tRNA molecules and defects in the processing of 35S rRNA [66]. In the human RNase P holoenzyme, Rpp20 is a component of the “finger” module; together with Rpp25 and Pop1, it stabilizes the C domain of *RPPH1* [4].

Several studies have revealed potential interactors of Rpp20, including SMN proteins (survival motor neuron), Hsp27 (heat shock protein 27) and KIAA0065 (zinc finger protein 33A) with the latter having a, so far, unknown role [60]. SMN proteins are correlated with neurodegenerative disorders, such as mutations in the human *SMN1* gene, which are linked to spinal muscular atrophy (SMA). SMN has a broad interactome, including Gemin proteins, Sm, Sm-like (Lsm) proteins and fibrillarin, which are involved in the assembly of snRNPs or play a role in rRNA processing. Co-immunoprecipitation experiments have shown that SMN also interacts with Rpp20 in humans, as well as in *Drosophila* [67]. While immunofluorescence analysis showed that, upon stress conditions (heat shock and UV irradiation), both proteins accumulate and co-localize in punctuated cytoplasmic structures known as stress granules, the exact role of both proteins’ accumulation in the granules is still elusive [67].

Yeast two hybrid screening but also pull-down assays using Rpp20 demonstrated a strong interaction with Hsp27, an ATP-independent chaperone that is constitutively expressed and upregulated by oxidative stress during aging and in cancers and protein deposition diseases [68]. This chaperone is also detected during RNase P holoenzyme purification from HeLa cells. However, when increasing the stringency of the washing steps, Hsp27 dissociates from the complex, while RNase P retains its activity. Interestingly, Hsp27, although not necessary for activity, when added to the RNase P reaction mixtures, it could stimulate RNase P activity [60].

### 3.4. Rpp21

In humans, the *RPP21* gene is mapped to chromosome 6p22.1 and resides in the class I gene cluster of the major histocompatibility complex (MHC). The protein is predominantly localized in the nucleoplasm but is also observed in nucleoli and Cajal bodies when expressed at high levels [69]. Rpp21 is a component of the Rpp21–Rpp29–Rpp38 heterotrimer that forms the “wrist” module of RNase P and stabilizes the connection between the C domain and the S domain of *RPPH1* [4]. Interestingly, Rpp21 together with Rpp29 and *RPPH1* can be recruited to double stranded DNA breaks (DSBs) to facilitate homology directed repair (HDR; discussed in Section 4.3) [50]. Finally, it was reported that Rpp21 colocalizes with histone H3.3 at transcriptionally active sites; however, its role in this case has not been investigated further [51].

### 3.5. Rpp25

Human Rpp25 is encoded by the *RPP25* gene, which is mapped to the locus ch15q24.2. Like Rpp20, Rpp25 is a member of the Alba-like superfamily, but without any known ATPase activity and is mainly localized in the nucleolus [65]. As mentioned above, Rpp25, together with Pop1 and Rpp20, form the “finger” module. Biochemical and chromatin immunoprecipitation studies showed that RNAi knockdown of Rpp25 leads to the transcription inhibition of genes encoding for 5S rRNA, rRNAs and tRNAs [49,70]. Interestingly, the knockdown of Rpp25 prevents the assembly of transcription initiation complexes through abolished or reduced recruitment of RPC6 and the RPC7 (DNA-directed RNA polymerase III subunit 6 and 7, respectively) [70]. Similarly, rescue experiments showed restored transcription levels of both tRNA and rRNA genes. Moreover, ChIP analysis revealed that the binding of Rpp25 to rDNA loci is independent of that of other protein subunits of RNase P and Pol I, such as Rpp20, which binds on promoter regions of 18S and 28S rDNA [49]. The binding of Rpp25 and Rpp20 in chromatin is more likely performed in a cell cycle–dependent manner and in agreement with the observed cell cycle–dependent regulation of rRNA genes transcription. It appears that Rpp25 binds on rDNA genetic loci during the late G1/S phase, an observation which is in good agreement with the increased transcription by Pol I, while Rpp20 is recruited to rDNA during the early G1 phase. Even though structural data showed that Rpp20 and Rpp25 strongly interact with each other, it is worth mentioning that their binding on chromatin occurs independently of each other [49].

**Figure 3:**
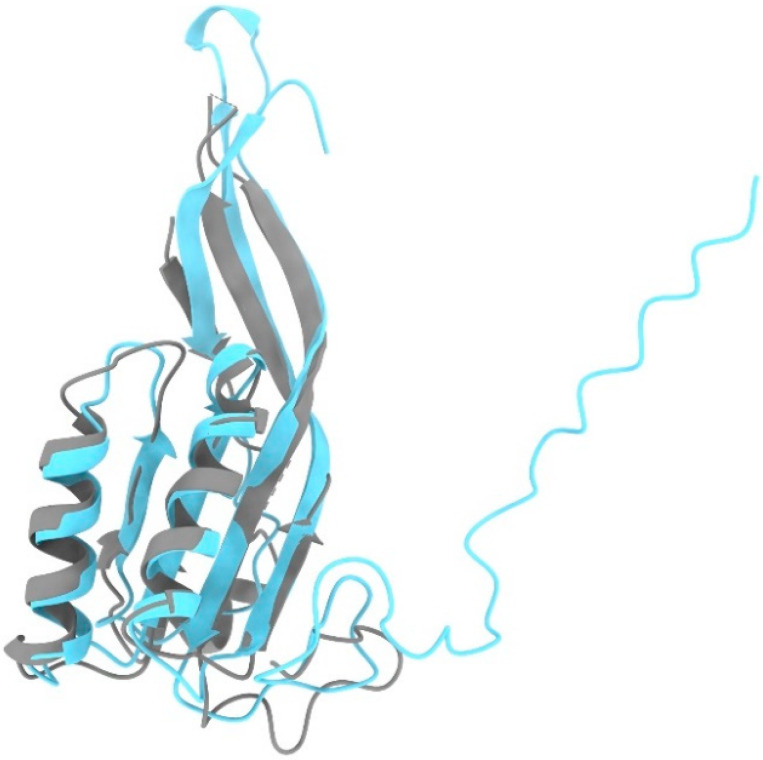
Superposition of the RPP25 Cryo-EM structure (grey, PDB ID: 6AHU) with the RPP25-like structural prediction model obtained from the AlphaFold database (light blue, Uniprot ID: Q8N5L8). The comparison was visualized using the Matchmaker tool in ChimeraX v1.2 [35].

Interestingly, a paralogue gene coding for Rpp25-like protein exists in vertebrates; however, its biological role is unknown. A comparison of the structure of Rpp25 from the human holoenzyme to the predicted structure of the Rpp25-like protein, which is deposited in the AlphaFold-Protein Structure Database, reveals (reported herein) a conformation very similar to that of Rpp25 [71]. This observation implies that Rpp25-like protein may serve as an alternative protein subunit of RNase P under specific conditions (Figure 3).

### 3.6. Rpp29

Human *RPP29* gene is mapped to chromosome 19q12, and the Rpp29 protein is localized in the nucleolus but also in Cajal bodies [58]. In the RNase P holoenzyme, hRpp29 interacts with hRpp21 and hRpp38 (“wrist” module) and forms the bridge between the C domain and S domain of *RPPH1*. Of note, Rpp29, except for its participation in DDR (discussed in Section 4.3), it also co-localizes with histone H3.3. It was shown that it can interact directly with the 69 N-terminal amino acids of H3.3 as well as H2B [51,52]. H3.3 is a conserved H3 type histone that is produced independently of the cell cycle; its mRNA is polyadenylated, unlike other histone mRNAs, an observation which implies a more stringent regulation [72]. Rpp29 represses the incorporation of H3.3 at euchromatinic regions via regulation of the post-translational modifications of the N-terminus and represses mRNA and protein expression. The dynamic nature of the interaction suggests that Rpp29 may represent a potential epigenetic modulator. Moreover, it was reported that, apart from Rpp29, fibrillarin and RPL23a are also found in the complex with histone H3.3, suggesting that these two proteins may be members of the Rpp29 interactome [51].

### 3.7. Rpp30

Rpp30 is the most well-studied protein subunit; in humans, it is encoded by the (essential for viability) *RPP30* gene, which maps to locus ch10q23.31. Given its role in RNase P/MRP complexes, Rpp30 is probably localized both in the nucleoplasm and nucleolus, a notion that awaits experimental confirmation [73]. Several knockout experiments performed in different organisms emphasize the importance of Rpp30. Rpp1, an *S. cerevisiae* Rpp30 homologue, is characterized as an essential protein subunit. Depletion of the Rpp1 protein leads to accumulation of unprocessed pre-tRNAs at both 5′ and 3′ ends, but also to defective rRNA processing at multiple sites (as a common protein subunit of the RNase MRP holoenzyme) [74]. In *A. thaliana*, the Gametophyte Defective 1 (GAF1) gene, which encodes the Rpp30 protein subunit of RNases P/MRP, is essential and plays an important role during gametogenesis [75]. In *Drosophila*, Rpp30 is also essential for viability; mutations are linked with sterility [53,54]. In addition, mutated Rpp30 leads to the activation of the DNA damage checkpoint in a Chk2-dependent manner. In this case, the PCNA (proliferating cell nuclear antigen) is not associated with DNA (in Rpp30 mutant germline cells) indicating a possible collapse of the replication forks because of the replication stress, due to the collision of stalled RNA pol III with DNA polymerase at tRNA genes sites. Additionally, Rpp30 mutant ovaries have a substantially lower abundance of piRNAs, which protect against transposons [54]. This effect is mainly attributed to alterations in the transcription regulation of the piRNA cluster, and not to inefficient piRNA biogenesis. Moreover, in Rpp30 mutant mice, piRNA repression is also observed most likely through the loss of H3K9 trimethylation in the chromatin surrounding the tRNA genes which, in turn, can affect major piRNA clusters found in close proximity to tRNA gene clusters. Finally, a recent study showed that the production of tRNA-derived fragments (tRFs) is also affected in Rpp30 mutant flies. More specifically, it was observed a significant increase in tRF-5s, i-tRFs and tRF-3s, accompanied with a decrease in tRF-1s, which derive from the 3′ trailer of pre-tRNAs [55]. The latter may be the result of the reduced efficiency of the 3′ end tRNA maturation since it is known that 5′ leader sequence removal by RNase P enhances the RNase Z activity at the 3′ end [76]. In addition, the abundance of mitochondrial tRFs is also increased substantially in the ovaries of mutant flies, indicating a possible involvement in the regulation of mt-tRF biogenesis [55]. Interestingly, two distinct subunits of Rpp30 were found in the human RNase P holoenzyme, in close contact to Pop5, Rpp14 and Rpp40 (forming the “palm” module), as well as domains of the *RPPH1* essential for catalysis, suggesting that the loss of Rpp30 may introduce substantial instability to the RNP (Figure 1) [5].

### 3.8. Rpp38

The human *RPP38* gene is mapped to chromosome 10p13. Similar to Rpp29, Rpp38 is localized in the nucleolus but also in Cajal bodies [58]. The structure of the human holoenzyme suggests that Rpp38 forms a heterotrimer with Rpp21 and Rpp29, and that Rpp38 interacts with Rpp21 to stabilize the *RPPH1* RNA through recognition of the K-turn (kink-turn) of the S domain [77]. Interestingly, hRpp38 has been identified as the main component, along with Pop1, Rpp25 and Rpp30, of the Th/To autoantigen present in the sera of patients suffering from systemic sclerosis scleroderma (SSc) [48]. Recently, it was reported that the Rpp38 epitope could increase sensitivity in the detection of anti-Th/To autoantibodies, enhancing the serological diagnosis of SSc [56]. In addition, the knockdown of Rpp38 leads to a reduction in the activity of RNase P and inhibition of expression of Rpp21, Rpp25, Rpp29, Pop5, as well as lamins A and C [19,78,79]. The above results suggest that under specific conditions, Rpp38 may dissociate from the holoenzyme, and its expression may affect the expression of other important protein subunits of RNase P.

### 3.9. Rpp40

hRpp40 is encoded by *RPP40,* which maps to locus chr6p25.1. It is considered a newly acquired protein, based on the absence of a Rpp40 homologue from archaeal and yeast holoenzymes (Figure 2). The localization of Rpp40 has not been experimentally validated, but as in the case of Rpp30, it is believed that it is localized in the nucleoplasm and nucleoli [73]. Based on the three-dimensional structural information of human RNase P, Rpp40 interacts with the C domain of *RPPH1* through extensive contacts with stems P1, P2, and P19. Stem P9 in yeast RNase P is shorter than that in humans, and this highlights the role of RPP40 in stabilizing the coaxially stacked helical core of the C domain. The N-terminus of Rpp40 mainly interacts with Rpp14 but also fits into the center of the Pop5-Rpp14-(Rpp30)_2_ tetramer, making contacts with Pop5 and one Rpp30 protein subunit. On the other hand, Rpp40 is connected to the “wrist” module through the interactions between Rpp29 and the other Rpp30 molecule [4]. So far, the Rpp40 protein has not been correlated with a non-canonical function.

### 3.10. Pop1

Pop1 is the largest protein subunit of the human RNase P holoenzyme, and its gene is mapped to chromosome 8q22.2. Immunofluorescence experiments showed that hPop1 is localized in the nucleolus and nucleoplasm [80]. The Pop1 protein replaces important RNA elements of RNA subunit of human RNase P holoenzyme, such as linker L5–15 and stem P5 in archaeal RNP. The N-terminus of Pop1 interacts with the Pop5 protein, connecting the “finger” and “palm” modules, while the C-terminus of Pop1 mediates interactions with *RPPH1* RNA via extensive interactions with the C domain, essentially embracing the C domain from one side as well as with the heterodimer Rpp20–Rpp25 [4]. In yeast, the *POP1* gene is essential for viability. Mutations can cause defects in pre-rRNA and pre-tRNA processing [81]. Moreover, yeast Pop1 protein has been found in the complex with Pop6–Pop7 in the RNP of telomerase. However, the exact role of Pop1 in this complex has not yet been defined [82,83]. hPop1 has been detected at histone H3.3 deposition sites along with Rpp29; however, hPop1 recruitment requires the region to be transcriptionally active. Furthermore, the silencing of Pop1 reduces the transcription rate at a transgene array that recruits H3.3 upon activation, but contrary to Rpp29, no direct interactions between Pop1 and H3.3 have been reported. The presence of Rpp21, Rpp29 and Pop1 at these sites could suggest the presence of a novel RNase P variant that has not been identified so far [51]. Interestingly, Pop1 was isolated as an autoantigen in patients suffering from connective tissue diseases, suggesting possible additional roles for Pop1 [80]. Finally, recent clinical reports showed that patients with mutations in the *POP1* gene exhibited anauxetic dysplasia 2 (ANXD2), characterized by extremely short stature [84].

## 4. Sub-Complexes of RNase P Protein Subunits

### 4.1. Rpp20 and Rpp25 Contribution to RNP Evolution

As mentioned before, Rpp20 and Rpp25 proteins belong to the Alba-like superfamily, a group of multifunctional proteins participating in various processes, such as RNA stability and metabolism, genome organization, and transcriptional and translational regulation [85]. Rpp20 and Rpp25 also participate as subunits of RNase MRP and, interestingly, consist of genuine protein subunits of the active yeast telomerase [86]. The integration of Rpp20 and Rpp25 into three important RNPs in eukaryotes, taken together with the ability of the Alba-like family to bind RNA and DNA, suggests that both proteins participated in key evolutionary events toward the formation of the contemporary RNPs, but also may have acquired new roles and functions. Extensive structural characterization highlighted the importance of the acquisition of eukaryotic Rpp20/Rpp25 during the evolutionary divergence of the RNA subunits. The two proteins bind to the specific P3 subdomain of all RNA subunits, a conserved feature found in all three RNP complexes (RNase P/MRP/Telomerase) [4,86,87]. The human RNase P holoenzyme structure as well as the crystal structure of human Rpp20–Rpp25 proteins in complex with the *RMRP* (RNA component of RNase MRP) confirmed their heterodimerization and binding to the P3 domain [87]. A similar behavior is observed between the homologues Pop6/Pop7 and Pop1 in yeast telomerase, which bind to a P3-like domain in the RNA moiety of telomerase, a structurally unrelated RNA, compared to *RPPH1* or *RMRP* [83]. Although the RNA subunit from prokaryotes contains the P3 RNA domain consisting of one helix, studies in both yeast and human showed that the P3 stem has a more complex structure (longer and larger internal loops), and its appearance was accompanied by the increased complexity of protein subunits [88,89]. The specific binding of Rpp20 and Rpp25 and P3 stem stabilization may play a role to accurate and timely holoenzyme assembly or could confer in substrate specificity. This is exemplified in yeast, where the deletion of the P3 hairpin of *RMRP* abolishes Pop6 binding and leads to reduced survival. In contrast, deletion or mutations at the end of the P3 domain that is recognized by Pop7 does not cause any noticeable defect [90,91,92].

### 4.2. Rpp20–Rpp25–Pop1–Pop4 Couple Transcription of RPR with RNase P Holoenzyme assembly

Arthropods, such as *Drosophila*, do not encode a distinct and recognizable gene that is dedicated to the production of the RNase P RNA subunit (RPR). Surprisingly, the RNA component of RNase P in these species is produced as part of intron 2 during the transcription of the *ATPsynC* gene. As the intron is transcribed, the RNase P subunits Pop1, Pop4 (homologue of hRpp29), Rpp25 and Rpp20 bind to the RPR and protect it from exonucleolytic activity of the Rat1/Xrn2 exonuclease and the nuclear RNA exosome. The knockdown of each of these protein subunits causes decreased abundance of RPR. On the other hand, knocking down Pop5, Rpp14, Rpp21 or Rpp30 does not significantly affect the expression levels of RPR. The role of the Rpp20–Rpp25–Pop1–Pop4 subcomplex in the production of RPR is essential and highlights the importance of protein subunit interactions for the assembly of the RNase P holoenzyme, which is coupled to RPR transcription [93].

### 4.3. Rpp21–Rpp29 and Their Role in RNase P Activity and Beyond

The addition of hRpp21 and hRpp29 during in vitro reconstitution assays can support RNase P activity in the presence of *RPPH1* RNA under specific conditions (low ionic strength and neutral pH) [94]. The same ability characterizes their archaeal homologues in similar experiments [32]. Based on the recent structural data, the observed activities could be explained by the fact that both proteins may stabilize the S domain of *RPPH1* and that Rpp29 represents the bridge between the C and the S domains [4]. In addition, hRpp29 can bind the bacterial M1 RNA and enhance its cleavage of a pre-tRNA substrate *in vitro*, suggesting that Rpp29 can provide the necessary environment for pre-tRNA binding, recognition and catalysis [94,95]. Further studies demonstrated that siRNA targeting of either Rpp21 or Rpp29 in HeLa cells lead to significant reduction in 5S rRNA and 45S pre-rRNA synthesis [49]. In addition to the previous results, hRpp21 and hRpp29 can participate in the DNA damage response through homology-directed repair (HDR), independently of their participation in RNase P [50]. More specifically, Rpp29 knockdown experiments in U2OS cells, prior to exposure to ionizing radiation (IR), led to accumulation of damaged DNA as well as the phosphorylation of specific markers, such as RPA2 (Replication Protein A2), that mediate in double-strand breaks (DSBs). Rpp29-depleted cells directly affected the HDR, while having no significant effect on non-homologous end joining (NHEJ) [50]. Interestingly, Rpp21 and Rpp29 are recruited to DNA damage sites though binding, on one end with poly ADP-ribose moieties and on the other, through interaction with the catalytic *RPPH1* RNA subunit, implying that possible interactions with the PARP family cannot be excluded.

## 5. Human Mitochondrial RNase P

The nature of RNase P from organelles, such as mitochondria and chloroplasts, puzzled the field for many years, with many reports suggesting that the holoenzymes were either very proteinaceous with an inaccessible RNA subunit or that the RNA subunit was dispensable for activity as suggested from experiments showing RNase P activity resistant to treatment with ribonucleases [96,97]. Surprisingly, the human mt-RNase P is a protein-only holoenzyme consisting of MRPP1, MRPP2 and MRPP3 protein subunits. Although MRPP3 is the catalytic subunit, MRPP1 and MRPP2 are required for mt-RNase P activity. Interestingly, MRPP1 and 2 (but not MRPP3) subunits exhibit distinct enzymatic activities, beyond the context of the holoenzyme. MRPP1 is an RNA-methyltransferase of mitochondrial tRNAs responsible for the introduction of m^1^A9 and m^1^G9 methyl group modifications, and MRPP2 is a mitochondrial dehydrogenase involved in fatty acid, branched-chain amino acid and neuroactive steroid metabolism [98,99]. Moreover, MRPP1 and MRPP2 are involved in the translation of the respiratory chain complexes through regulation of mitochondrial lncRNAs expression and ribosome assembly [100].

**Figure 4:**
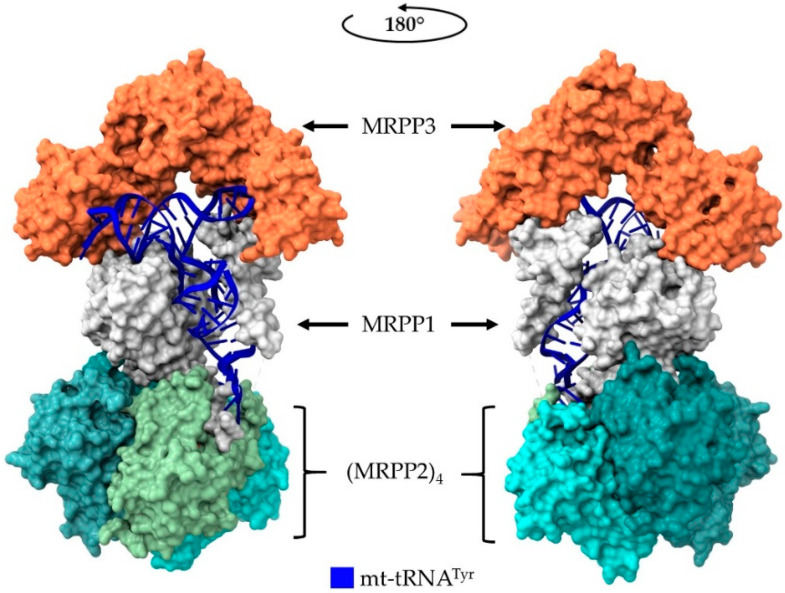
Cryo-EM structure of human mt-RNase P bound to mt-tRNA^Tyr^ as it was deposited in PDB (PDB ID: 7ONU). The structures were visualized using ChimeraX v1.2 [35].

The structures of MRPP1, MRPP2 and MRPP3, determined individually and combined with low resolution SAXS analysis of the holoenzyme, gave a first description on the stoichiometry and the architecture of the human mt-RNase P [10,45,101,102]. A recent Cryo-EM structure of human mt-RNase P in complex with mitochondrial pre-tRNA-Tyr that was recently published (PDB ID: 7ONU) provides a more detailed picture of mt-RNase P holoenzyme’s composition, function and substrate recognition (Figure 4) [44]. Of note, the 3.0 Å resolution structure reveals a heteroexamer, with MRPP2 forming a tetramer that interacts with the anticodon stem-loop of tRNA, which subsequently interacts with a monomeric MRPP1 protein subunit. MRPP1 wraps around the tRNA molecule and interacts with the MRPP3 catalytic subunit, which in turn, interacts with the tRNA “elbow” and the acceptor stem. However, pre-tRNA-Tyr contains, like most mitochondrial tRNAs, shorter non-canonical D- and T-arms and does not interact with MRPP3 through the U/G19 and C56, which are conserved only in the cytoplasmic tRNAs. The extensive interactions of MRPP1 and MRPP2 that extend along the tRNA body differ from what has been described for the PRORPs from plants and archaeal HARPs since the latter exhibit interactions with the tRNA “elbow” and the acceptor stem [39,40]. This unique structural organization and extensive protection of the tRNA substrate explains how the MRPP1/MRPP2 complex stabilizes the conformation of the structurally peculiar mitochondrial pre-tRNAs not only for 5′ end cleavage, but also for m^1^G/A methylation at position 9 of mitochondrial tRNAs, the efficient 3′ end maturation, and finally, the 3′ CCA addition [10,44,103].

Given that the mitochondrial RNA maturation process obeys the tRNA-punctuation model, it is possible that ablation of either subunit of the mt-RNase P complex could have excessive effects, leading to lethality [104]. As expected, knocking-out MRPP3 in mice and its corresponding homologue in *Drosophila* (*mldr*; Mulder) leads to accumulation of precursor transcripts and is lethal during early development. The same effect is also observed after knocking down or completely inactivating the MRPP1 and MRPP2 homologues in *Drosophila* (*rswl*; Roswell and *scu*; Scully, respectively). Knocking out MRPP1 and MRPP2 in a mammalian animal model has not yet been tested; however, knocking down MRPP1 and MRPP2 in HeLa and U2OS cell cultures disrupts the proper maturation of mitochondrial RNA and leads to an increased abundance of higher molecular weight transcripts [98,105]. Mutations in the gene coding for MRPP2 cause HSD10 mitochondrial disease, which is characterized by severe neurological deficiencies and severely decreased life-expectancy [106]. Moreover, recessive mutations in the gene coding MRPP1 can lead to infantile death at approximately 5 months after birth [107]. A viable conditional *MRPP3* knockout mouse model in heart and skeletal muscle tissues was developed, but the mutants exhibited short lifespans and succumbed at approximately 11 weeks due to cardiomyopathy [108]. Isolated heart mitochondria from the same mouse model revealed that although the rate of transcription was elevated, the translation rate was significantly reduced. Despite the increased transcription rate, longer unprocessed transcripts were produced that could not be processed further. Moreover, the mitochondria exhibited extreme OXPHOS defects, due to the impaired mitochondrial protein synthesis, which resulted in the reduced production of functional respiratory complexes. Even though mitochondria possess a functional RNase Z, the enzyme’s activity was blocked by the presence of unprocessed tRNA 5′ ends. Taken together, the above observations indicate that mt-RNase P-mediated maturation of tRNAs precedes the 3′ end processing, such as in nucleus. Recently, it was shown that mt-tRNAs require the presence of the MRPP1/MRPP2 complex for efficient 3′ processing, suggesting that the presence of mutations or depletion of either protein may lead to disruption of both 5′ and 3′ processing [10]. Finally, knocking out each individual subunit in *Drosophila* larval neuroblasts results in mitochondrial swelling. The complete absence of each subunit differentially affects the 5′ and 3′processing of tRNAs, but also of non-canonical junctions, depending possibly on their upstream RNA content. Additionally, the effect on the steady-state levels of transcripts produced from the same polycistronic transcript differ upon knocking out each mt-RNase P subunit. These discrepancies possibly stem from the fact that other ribonucleases may have moonlighting mitochondrial RNA processing activity [109].

## 6. Conclusions

**Figure 5:**
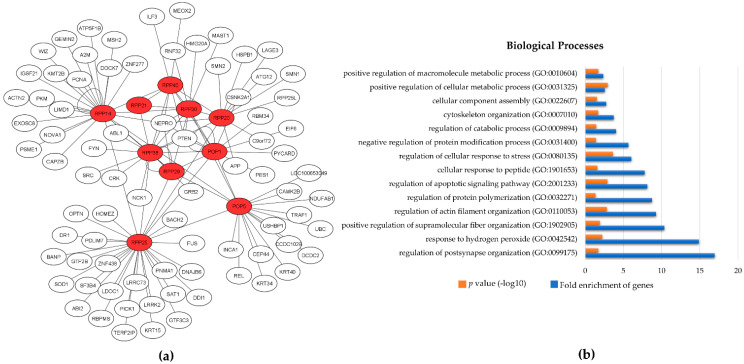
(**a**) Protein–protein interaction network of RNase P subunits; (**b**) gene ontology enrichment analysis on the RNase P subunits’ interactants. The protein–protein interactions of each RNase P protein subunit were retrieved from PICKLE (Protein InteraCtion KnowLedgebasE) and illustrated, using Cytoscape v3.8.2 [111,112]. Gene ontology enrichment analysis was performed, using the PANTHER classification system, and P-values were subjected to Bonferroni correction for multiple testing [113].

Almost 40 years after its original discovery and characterization, RNase P remains an appealing model to study structural and functional attributes during the transition from an RNA to an RNP, or even protein, world. The multi-subunit protein composition of both the nuclear and mt-RNase P holoenzymes suggests that proteins with possibly pre-existing function were acquired during evolution and some of them still serve important alternative cellular roles, either as individual components or in combination with other proteins. The fact that many protein subunits act as molecular “patches” to cover for the structural peculiarities or deficiencies of RNA is an important feature that possibly could reflect the putative structural heterogeneity of the holoenzyme under specific conditions (i.e., during developmental stages). The existence of three RNase P RNA-like transcripts in mice (*Rprl1-3*), which are differentially expressed in various tissues, suggests that possible heterogeneous pools of RNase P RNPs may exist [7]. In support of this notion, it was proposed that the human RNase P holoenzyme composition may vary across tissues or under specific conditions or during the progression of diseases [20]. Given that similar structural variability and heterogeneity was reported for ribosomes, the notion that RNase P holoenzymes could exhibit similar biochemical character remains a possibility and an open question to address [110]. Such variability could account for altered tRNA and rRNA production rates and could affect transcription and translation levels, thereby providing new means of gene expression regulation. In addition, a closer look at the putative interactome of the protein subunits that we provide herein clearly shows that several proteins could transiently interact with each of the RNase P protein subunits, thus forming an expanded and dynamic network (Figure 5a). Gene ontology enrichment analysis of the putative protein subunits interactants shows that RNase P could provide a major regulatory hub (Figure 5b). Interactions of the protein subunits with important proteins, such as the onco-suppressor PTEN or Hsp27, may play a role in altering the functional rigidity of RNase P and could fine-tune alternative cellular biological functions.

Notably, eukaryotes possess also RNase MRP endoribonuclease, which is related to RNase P; in humans, both RNPs share 9 out of 10 protein subunits (except from Rpp21). Although RNase MRP was initially characterized as a nuclease that cleaves the primers for DNA replication in mitochondria, later, it was shown that it is involved in pre-rRNA processing by cleaving the A3 site in the internal transcribed spacer 1 (ITS1). Interestingly, individual reports have linked RNase P and/or MRP activity with the cleavage of specific mRNAs, either in their 5′UTR (as in the case of Cyclin B2 mRNA) or through interactions with YTHDF2 (YTH N6-Methyladenosine RNA Binding Protein 2) and HRSP12 (2-iminobutanoate/2-iminopropanoate deaminase), which are recruited on m6A modification sites to regulate mRNA cleavage [114,115]. In addition, as described previously, yeast RNase P shares three subunits with the active yeast telomerase (yeast homologues of Rpp20, Rpp25 and Pop1) with a role in the stability and transportation of the telomerase RNA component (TERC) from the cytoplasm to the nucleus [82,83]. Finally, the observation that protein subunits, which are shared among the RNase P, RNase MRP and telomerase RNPs, possess DEAD-like and ATPase motifs, also found in helicases, chromatin modifiers and remodelers, suggests that they may have alternative modulatory roles beyond RNA maturation and metabolism. The advantage of new genome editing tools provides a unique opportunity to study the contribution of each subunit in cell lines and animal models and to assess the importance and dispensability of each subunit for the recombinant holoenzymes. In addition, studies based on the genomic ablation or addition of specific RNase P subunits may shed light on their alternative roles inside cells and their possible contribution to gene expression regulation at the post-transcriptional level.

## Figures and Tables

**Table 1:** RNase P RNP subunits across the domains of life.

RNase P	Bacteria (*E. coli*)	Archaea (*M. jannaschii*)	Yeast (Nuclear)	Human (Nuclear)
RNA subunit	M1 RNA or P RNA (377 nt)	RPR (252 nt)	RPR1 (369 nt)	RPPH1 (341 nt)
Protein Subunits *	RnpA/ C5/P protein (13.8 kDa)			
		Pop5 (15.9 kDa)	Pop5 (19.6 kDa)	Pop5 (18.8 kDa)
		Rpp29 (10.9 kDa)	Pop4 (32.9 kDa)	Rpp29 (25.4 kDa)
		Rpp30 (27.3 kDa)	Rpp1 (32.2 kDa)	Rpp30 (29.3 kDa)
		Rpp21 (15.6 kDa)	Rpr2 (16.3 kDa)	Rpp21 (17.6 kDa)
		L7Ae/Rpp38 (12.7 kDa)	Pop3 (22.6 kDa)	Rpp38 (31.8 kDa)
			Pop1 (100.4 kDa)	Pop1 (114.7 kDa)
			Pop6 (18.2 kDa)	Rpp20 (15.7 kDa)
			Pop7/Rpp2 (15.8 kDa)	Rpp25 (20.6 kDa)
				Rpp14 (13.7 kDa)
				Rpp40 (41.8 kDa)
			Pop8 (15.5 kDa)	

* Proteins within the same row share sequence homology.

**Table 2:** RNase P RNP subunits and additional roles.

Subunits	Structure	Additional Roles	References
Rpp14	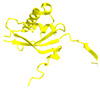	Exhibits 3′→5′ exoribonucleases activity	[46]
Pop5	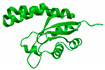	Unknown	
Rpp20	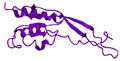	Exhibits ATPase activity, promotes transcription by RNA Pol I and III, binds to chromatin of rDNA genes, Th/To autoantigen	[19,47,48,49]
Rpp21	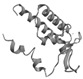	Promotes DNA repair through HDR	[50]
Rpp25	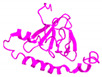	Promotes transcription by RNA Pol I and III, binds to chromatin of rDNA genes, Th/To autoantigen	[19,48,49]
Rpp29	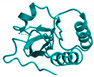	Promotes DNA repair through HDR, nucleosome remodeling	[50,51,52]
Rpp30	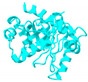	Affects piRNAs transcription and tRF biogenesis	[53,54,55]
Rpp38	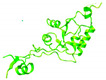	Th/To autoantigen	[56]
Rpp40	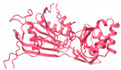	Unknown	
Pop1	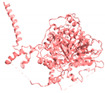	Transcription, Th/To autoantigen	[48,51]
